# Cytokine Gene Expression Alterations in Human Macrophages Infected by *Leishmania major*

**DOI:** 10.22074/cellj.2021.6524

**Published:** 2020-04-22

**Authors:** Khodaberdi Kalavi, Ogholniaz Jorjani, Mohammad Ali Faghihi, Seyed Javad Mowla

**Affiliations:** 1 Department of Molecular Genetics, Faculty of Biological Sciences, Tarbiat Modares University, Tehran, Iran; 2 Department of Laboratory Sciences, School of Allied Medical Sciences, Golestan University of Medical Sciences, Gorgan, Iran; 3 Department of Medical Genetics, Shiraz University of Medical Sciences, Shiraz, Iran; 4 Department of Psychiatry and Behavioral Sciences, School of Medicine, University of Miami, FL, USA

**Keywords:** Chemokines, Cytokines, *Leishmania major*, Macrophages, RNA Sequencing

## Abstract

**Objective:**

Leishmaniasis is caused by members of the *Leishmania* species and constitute a group of infective diseases that range from cutaneous lesions to lethal visceral forms. In infected persons, macrophages recognize and eliminate the parasites via phagocytosis. In order to change a hostile environment into an environment adequate for survival and reproduction, the engulfed Leishmania species needs to modulate the function of its host macrophage. The expression patterns of cytokine genes such as interleukin-12 (*IL-12*), tumour necrosis factor-alpha (*TNF-α*), *IL-1*, and interferon-gamma (*IFNγ*) represent the immune response. In this study, we employed an RNA-seq approach for human monocyte-derived macrophages infected with *Leishmania major (L. major)* to decipher cytokine gene expression alterations in host macrophages.

**Materials and Methods:**

In this descriptive study, human monocytes were isolated by magnetic activated cell sorting (MACS) and cultured in the presence of monocyte colony stimulating factor (M-CSF) to obtain the macrophages. Monocyte-derived macrophages were then co-cultured with metacyclic promastigotes of *L. major* for 4 hours. RNA isolation was performed using TRIzol reagent. RNA sequencing was performed using the Illumina sequencing platforms. Gene expression analysis was performed using a Bioconductor DESeq2 package.

**Results:**

Our data revealed significant changes in immune response gene expressions in macrophages infected with L. major, with an up-regulation of cytokines and mostly down-regulation of their receptors.

**Conclusion:**

The obtained data could shed more light on the biology of *L. major* and how the host cell responds to leishmaniasis.

## Introduction

Leishmaniasis is a worldwide chronic inflammatory disease caused by *Leishmania* species. Leishmaniasis has cutaneous, mucocutaneous and lethal visceral forms. Macrophages phagocytize pathogens such as *Leishmania* species. However, when inside macrophages, the *Leishmania* species adapt and modulate the host microenvironment for better survival and reproduction. Understanding the means used by these pathogens to alter host defence mechanisms for intracellular survival and reproduction could help to define novel diagnostics and therapeutics for leishmaniosis (1).

The host response to infection is regulated by controlled production of cytokines. In mice and humans, resistance against many pathogens, including *Leishmania* species, is associated with T helper type 1 (Th-1) cell cytokine response that includes interleukin 12 (IL-12) and gamma interferon (IFN_Ɣ_). Susceptibility to infection is associated with production of the Th-2 cytokines IL- 4, IL-5, and IL-10 (1). High-throughput technology has made it possible to define and analyse large sets of genes or proteins that modulate in response to host-pathogen interactions. Recent studies that used techniques such as microarray have generated some data. However, the limitations due to hybridization have recently improved with next generation sequencing (NGS) technology (2). Accordingly, several *in vivo* and *in vitro* models of animal and human host cells have been developed, especially monocyte derived macrophages (MDMs).

*Leishmania* species alter cytokine production in infected host cells towards the pathogen’s benefit. Important altered cytokines include IL-12, TNF-α, IL- 1, and IFNɣ. Receptor mediated phagocytosis causes alterations such as suppressed production of IL-12. This cytokine immunologically transduces signals to produce and activate qualified Th-1 cells which, in turn, leads to IFNɣ production and natural killer (NK) cell proliferation and activation as the major source of IFNɣ (3). IFNɣ interacts with the IFNɣ receptor (IFNɣR) expressed on the macrophage surface and leads to pathogen disposal by host immune cells (4). In leishmaniasis, the aforementioned parameters are altered.

There are reports on early shifting of Th-2 cells where cytokines produced by these cells suppress the protective capacities of the immune system. IL-10, produced by this type of cell, is associated with both decreases in nitric oxide (NO), IFNɣ, and IL-12 gene expressions and inhibition of protein kinase C (PKC) in infected macrophages (5). In this direction, Leishmania species induce the regulatory T (Treg) cells to suppress antileishmania immune responses in cutaneous and visceral animal models and in human leishmaniasis (6). T-regs generally function as immune preventers against effector Th-1 cells and *Leishmania* parasites use this potential to escape from Th-1 related eradication. T-regs downregulate Th-1 related macrophage activity by secreting IL-10 and transforming growth factor beta (TGF-β) at the site of infection, resulting in changes that are favourable to the pathogen (7).

Chemokines, another specific type of cytokine, plays a crucial role in the anti-leishmaniasis immune response and are modulated by the parasite (8). Chemokines are small polypeptides that contain cysteine residues within the polypeptide chain (9).

Control of leishmaniasis strongly depends on an IL-12 driven Th-1 cell response and IFNɣ production that, in turn, causes facilitated recruitment of effector cells (macrophages, NK cells, CD4+ and CD8+ cytotoxic cells) to the site of infection (10). The chemokine production strategy is very important as it determines cellular recruitments and communications needed to establish a proper immune response. Chemokines act through binding to chemokine receptors, which leads to a variety of biological functions, such as directed cellular migration (11).

Here, we performed an RNA seq approach study using an Illumina sequencing platform to determine transcriptome changes in macrophages infected with *Leishmania major (L. major)*.

## Materials and Methods

### Study method

The present study was approved by the Ethics Committee of the Guilan University of Medical Sciences (Guilan, Iran, Ethical code: IR.Goums.1397.293). This was a descriptive study of RNA-seq data on human monocyte-derived macrophages infected with *L. major* to decipher gene expression alterations of the cytokines in host macrophages.

### Peripheral blood mononuclear cell preparation

Healthy donors donated blood at the Gorgan Blood Bank. The buffy coat fractions in the whole blood bags were used for separation of peripheral blood mononuclear cells (PBMCs). A 1:1 (v:v) phosphate-buffered saline (PBS) and the buffy coat sample were transferred into 50 ml sterile falcon tubes thatwhich contained an equal amount (v:v) of Ficoll solution (density 1.077 g/mL, Baharafshan, Iran). We obtained the separated PBMCs after 25 minutes of centrifugation at 300 g. The PBMC were then washed three times using a sterile PBS solution and were subdivided into fresh sterile tubes.

### Monocyte isolation

The magnetic activated cell sorting (MAC) method (Miltenyi Biotec, Germany) was used to obtain monocytes with a high purity.

### Macrophage preparation

After cell counts and adjustment to 1 250 000 cells/well, we cultured these cells in Roswell Park Memorial Institute (RPMI 1640) media (Gibco, USA) in sterile T25 (2×6 well) flasks supplemented with 10% fetal bovine serum (FBS, Sigma, USA), 1% penicillin/streptomycin (pen/ strep) and 20 ng/ml of monocyte colony stimulating factor (M-CSF, Miltenyi Biotec, Germany). This complex was incubated at 37˚C, with 5% CO_2_ under high moisture conditions for 7-9 days with media changes every two days.

### Parasite culture

MRHO/IR/75/ER (IR endemic) were cultured in RPMI 1640 and incubated (22-25˚C) for 3-6 days. Promastigotes were collected during the stationary phase, transferred to a sterile Ficoll tube and centrifuged (350 g) for purification.

### Macrophage-promastigote co-culture

We used two, 6-well T25 flasks to co-culture 5-7 promastigotes per macrophage in the presence of M-CSF for 4 hours. At the same time, and in parallel, 5-10 polystyrene latex particles (4.16 μl) per cell were used for phagocytosis by the macrophages over 4 hours. In parallel, one plate of uninfected macrophages was collected after 4 hours to be used as the control (9 replicates in 3 groups).

### RNA isolation

Total RNA was extracted using TRIzol (Invitrogen, USA) reagent and stored at -75˚C. A Nanodrop was used for 260/280 and 230/260 ratio assessments and 1% agarose gel electrophoresis was performed for determination of the 28S/18S ratio. The RNA integrity number (RIN) of total RNA was assessed using an Agilent 2100 Bioanalyzer system.

### cDNA synthesis and RNA-Seq

For transcriptome sequencing (SureSelect, Agilent, USA, 2017), cDNA libraries were prepared from the RNA obtained at two different time points (0 and 4 hours after infection). cDNA synthesis was performed using oligo dTs against a 3′ poly (A) tail according to the manufacturer’s instructions. Quality-controlled cDNA were sequenced using an Illumina RNA-Seq workflow method. Single-end reads and the resultant reads were arranged and trimmed. Transcripts were mapped against the human genome, hg38/GRCh38.

### Bioinformatics and statistical analyses

High quality sequencing data were analysed using our bioinformatic pipeline that consists of FastQC for quality controls, Trimmomatic for trimming, and Kallisto for pseudoalignment to the transcriptome. Data analysis was performed with a Bioconductor DESeq2 package for data normalization and DE analysis. P≤0.05 was considered for statistical significance and the 5 FC threshold based on log2 for deferential expression assays. For gene annotation, we used an online software program, the Biological Database Network (bioDBnet) and for log2 fold change conversion, we used a base 2 logarithm (Log2) calculator.

## Results

### Gene expression integrity assessments

**Fig.1:**
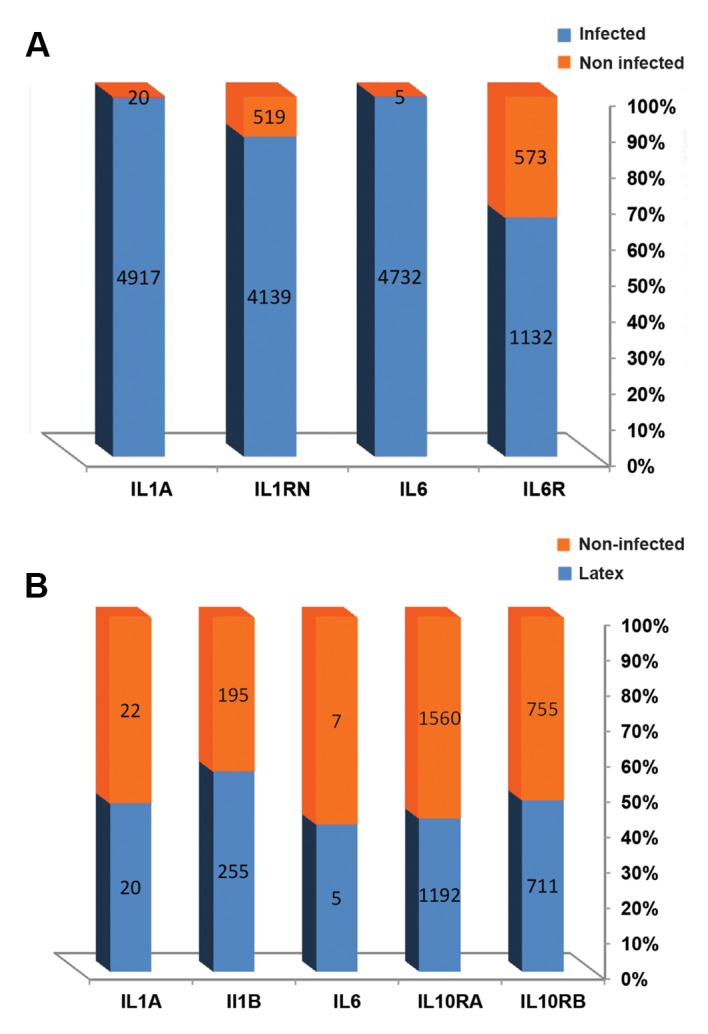
Gene expression pattern showing different expressions of noninfected samples compared to infected samples. As seen in the noninfected cells, the gene expression pattern is almost similar. In infected cells, the gene expression pattern is clearly altered. **A.** The different expression patterns for infected (grey column) and non-infected (red column) cells are shown. **B.** Non-infected cells exhibit a more similar gene expression pattern.

The majority of anti-leishmania effects on host cells occur after phagocytosis. For this reason, we assessed the test and control samples after phagocytosis. We used total RNAs from three repeated micro-bead latex particles ingested by the macrophages to determine the triggering power of inert particles on macrophage gene expression (phagocytosis effect). As shown in Figure 1A, B, the inert particles did not trigger transcriptome changes at 4 hours post-infection (4hpi), which indicated similar expression patterns as the non-infected macrophages.

### Cytokine gene expressions

**Fig.2:**
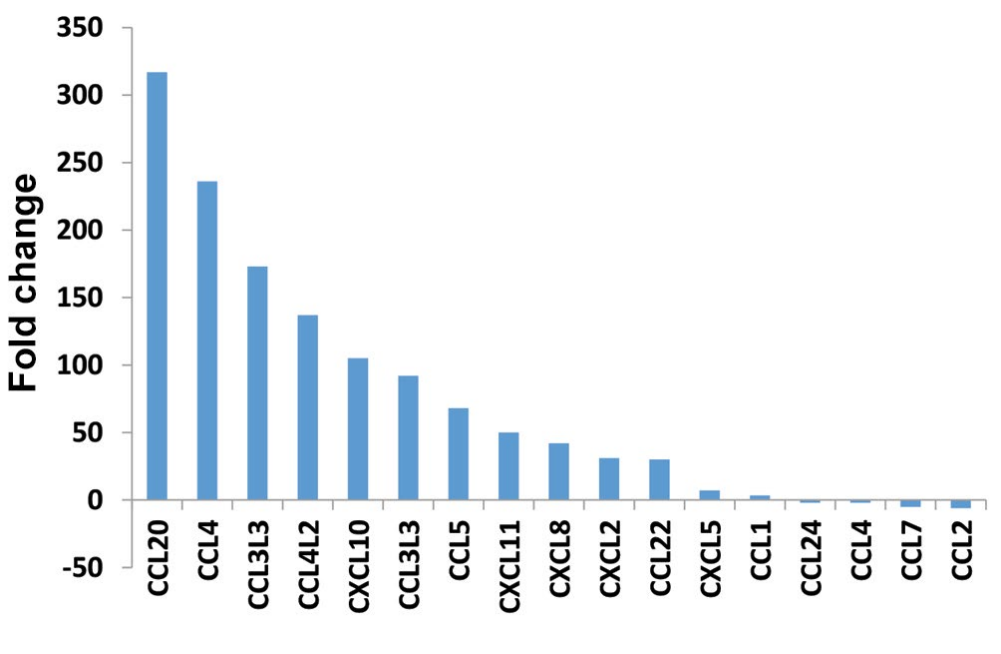
Expression levels of CC chemokine genes are higher than CxC chemokines. CCL2 and CCL7 chemokines were down-regulated. CCL2 seems to be one of the most important targets of *Leishmania major (L. major)* that is reprogrammed by this parasite. Most of the chemokine genes showed up-regulated expression patterns; thus, it may indicate the importance of cellular recruitments early infection time point.

Immune response and inflammatory cytokines *IL- 1a/b,* tumour necrosis factor alpha *(TNF-α)* and TNF superfamily genes *(IL-6, IL-2, IL-12, IFNs)* were upregulated with higher transcript reads expressed in the infected macrophages compared with the other samples (Table 1). Chemokines mostly up-regulated and only a few were down-regulated (Fig.2). *IL-1a, b,* TNF-α, and *IL-6s* all up-regulated as a result of pro-inflammatory stress caused by the pathogen (Table 2). *IL-27* was upregulated, whereas we observed down-regulation of *IL- 27*R. Some cytokines such as *IL-1, TNF-α* and the IFNs were up-regulated. In particular, *IFNɣ* was up-regulated. IFNs are produced by activated macrophages, T cells, and NK cells. IFNɣ pathway regulation is critical to lipopolysaccharide (LPS) induced and Toll-like receptor (TLR) related pathway responses.

**Table 1:** Cytokines and their receptor RNA sequence read counts


Cytokine	Latex ingested	Non- infected	Infected

IL-1A	22	20	4917
IL-1B	255	197	8772
IL-1R1	223	1064	1352
IL-1RN	413	526	4139
IL-2	0	0	18
IL-2RA	7	0	148
IL-2RG	568	338	817
IL-3RA	50	42	29
NFIL3	222	217	40
IL-4R	631	578	1168
IL-15	31	12	78
IL-6	5	7	4752
IL-6R	382	573	1132
IL-7R	86	591	1203
IL-10	6	13	47
IL-10RA	1192	1560	4412
IL-10RB	711	775	502
TNF	10	2.5	3114
TNFRSF12A	61	43	67
TNFRSF1B	2806	3900	4819
TNFRSF9	191	188	1543
TNFRSF1A	604	602	439
TNFSF13B	354	265	194
TNFAIP1	160	133	340
TNFAIP3	488	404	7490
TNFAIP8	480	475	3156
TNFAIP6	112	63	5474
TNFSF9	56	25	1033
IL-15RA	2	17	293


**Table 2:** Cytokines and their receptor fold changes


	Genes	Fold change

Interleukins and their receptors	IL-2	55 ↑
	IL-2RB	50 ↑
	IL-12A and B	18 and 117 ↑
	IL-6	678 ↑
	IL-18	4 ↑
	IL-10, IL-24	4 and18 ↑
	IL-23A, IL-27	268 and >100 ↑
	IL-1RN	8 ↑
	IL-15, IL-15RA	7 and 15 ↑
	IL-1A and B	33 and 55 ↑
TNFs and their receptors	TNFSF8	2 ↓
	TNFAIP3	17 ↑
	TNFAIP2	13 ↑
	TNFRSF9	8 ↑
	TNFRSF4	12 ↑
	TNFAIP8	6 ↑
	TNFSF1B	1.5 ↓
	TNF-α	176 ↑
IFNs and their receptors	IFNB1	55 ↑
	IFNL1	21 ↑
	IFNƔ	5 ↑
	IFNƔR1	3 ↓
	IFNAR1	1.5 ↓
	IFNAR2	1.5 ↓
CSFs	CSF1	19 ↑
	CSF2	142 ↑
	CSF3	67 ↑


TNF; Tumour necrosis factor, INF; Interferon, and CSF; Colony stimulating factor.

### Growth factors and chemokine cytokine type gene expressions

Growth factors, colony stimulating factors *(CSF) 1, CSF2* and *CSF3* were up-regulated in the infected cells (Table 2). Both *CSF-1RB* and *CSF-2R* were insignificantly down-regulated. *CSF-3R* was insignificantly up-regulated. Both *CCL2* and *CCL7* and their receptors were downregulated. *CCL2* and its receptors, *CCR2* and *CCR4*, act to recruit monocytes and macrophages to the site of infection. The majority of chemokines were significantly up-regulated (Fig.2).

## Discussion

The expression status of cytokines, as an indicator of the immune response, depends on the type of pathogen and its virulence factors, as well as the genetic background of the host. Thus the immune response to *L. major* continues from the moment it enters the body until it is processed by immune cells such as macrophages. During this process, *L. major* modifies the host defence mechanisms in its favour for better survival and transforms the target cell, such as the macrophage, into a safe environment for itself.

*Leishmania* species benefit from a variety of tools in achieving this goal. Most notably, the selection of specific receptors from the macrophage-level complement system (CR1,^3^) and modulation of TLRs to neutralize the highly lethal phagolysosomal system, disruption of exposure of antigens to T cells, and anti-apoptotic changes, especially those that occur with the production and function of cytokines. In fact, *Leishmania* species control host defence mechanisms by activating or suppressing them.

The expressions of many cytokines in the infected host cells are altered by these pathogens. Pro-inflammatory and anti-inflammatory cytokine changes have been the main focus of many reports on cytokine alterations in leishmaniasis (12). The most important of these cytokines are IL-12, IL-6, IL-23, IL-1 alpha and beta, IL-10, IL-18, IL-15, and IL-2, TNF-α, IFNƔ, and TGF-β.

IL-12 is one of the most important cytokines produced by infected macrophages that has a role in Th-1 cell response. It is reported to be suppressed in these aforementioned cells (13). As a key cytokine gene that enables host macrophages to kill or clean up pathogen, *IL-12* is highly expressed by macrophages. However, they lose this killing capability because of inhibitor of serine protease (ISP) produced by the parasite (14). *IL-12, IL-23*, and *IL-27* genes with structural and functional homology showed significant up-regulation in our study.

Pro-inflammatory cytokine genes *TNF-α, IL-1a, IL-1b, IL-6, IL-8* and *IFN_Ɣ_* were significantly up-regulated in our study, and reports of these types of cytokine genes were inconsistent. There is no RNA-seq-based report on this subject. Dillon et al. (15) reported an overall increase in the expression of immune and metabolic response genes, and the involvement of signalling pathways which supported our data. Therefore, because of the lack of such data, this study could be served as a reference for future studies.

In this study, despite some significant increases in the expression of cytokine receptor genes *IL-1R, TNF-Rs, IL- 6R, IL-15R,* and IL-2R*,* other receptors had low or even reduced expressions. There is a lack of RNA-seq studies on cytokine receptor genes in leishmaniasis.

In this study, *IL-2*, *IL-15* and their receptor genes had significant upregulation. These two cytokines have a similar function and are involved in the proliferation and survival of Th cells. In addition, the effect of *IL-2* gene expression on macrophage dysfunction with *IL-12* gene expression has been reported (16). This confirms a possible dual role of some of these molecules, which has been reported by Abdoli et al. (17) who assessed the dual role of *IL-10* and *TGF-β* genes in *L. major* species.

An association has been reported between *IL-10*, a suppressive cytokine gene expressed by Treg cells, and leishmaniasis. *IL-10* expression suppresses the Th-1 cellular response to leishmaniasis. In this study, we have observed increased expression of *IL-10* and its receptor genes, and partial upregulation of *TGF-β* and its receptor.

Chemokines and their receptors are another type of cytokine. They play an important role in the processes of directing immune cells to pathogens. *Leishmania* species also play a critical role by altering chemokine expression for their own benefit. Chemokines, on the other hand, carry immune cells to the site of infection or inflammation and perform other biological functions. For example, CCL2, despite attracting monocytes, macrophages, dendritic cells, and memory T cells, also exhibits antileishmania effects and may be one of the pathogen targets in the pathway of suppression. In the current study, *CCL2* had a six-fold decrease in expression. On the other hand, lipophosphoglycan (LPG), which is present on surface of *Leishmania* species has inhibitory effects on monocyte migration. *CCL2* induces secretion of molecules such as selectins, ICAM-1, and VCAM-1. In this study, there was a 21-fold increase in *ICAM-1* and a 15-fold increase in *ICAM-5* gene expression. *CCR2,4*, specific *CCL2* receptor genes, were not expressed in this study. *CCL3* and its receptor genes, *CCR1,5*, had increased expression. The *CCL7* chemokine gene had decreased expression in this study. This chemokine is reported to increase functionally at the site of infection and, while recruiting monocytes, shows anti-macrophage function effects (18). Since *CCR1,5* also acts as receptor genes for these chemokines, it seems that their function is not solely dependent on ligand-receptor binding because *CCL7* selectively binds to Th-2 cells and prevents them from acting. We observed a 2-fold increase in *IL-4* expression.

Other *CCL* and *CXCL* genes also had increased expressions in this study. Most of these chemokines are products of IFNɣ signalling pathways. *CXCL10 (IP10)*, which showed increased expression in this study, has antiangiogenic effects related to IL-12 activity associated to IFNɣ (19).

## Conclusion

We used an RNA-seq approach to decipher the pattern of gene expression alterations in early immune response and inflammatory pathways in macrophages infected with *L. major*. The obtained data could shed more light on the biology of *L. major* and the host cell response to infection. Our data also demonstrated up-regulation of some important pro-inflammatory cytokines during the early post-infection period.
